# Core Competencies of the Modern Geriatric Cardiologist: A Framework for Comprehensive Cardiovascular Care in Older Adults

**DOI:** 10.3390/jcm15020749

**Published:** 2026-01-16

**Authors:** Rémi Esser, Alejandro Mondragon, Marine Larbaneix, Marlène Esteban, Christine Farges, Sophie Nisse Durgeat, Olivier Maurou, Marc Harboun

**Affiliations:** 1 Cardiogeriatrics Department, Hôpital La Porte Verte, 78000 Versailles, France; alejandro.mondragon@lpv.univi.fr (A.M.); marine.larbaneix@lpv.univi.fr (M.L.); marlene.esteban@lpv.univi.fr (M.E.); christine.farges@lpv.univi.fr (C.F.); olivier.maurou@lpv.univi.fr (O.M.); marc.harboun@lpv.univi.fr (M.H.); 2 Medical Affairs, NP Medical, 33000 Bordeaux, France; sophie@satelia.eu

**Keywords:** heart failure, older adults, frailty, cardiogeriatrics, multidisciplinary care

## Abstract

**Background**: The rapid ageing of the cardiovascular population has profoundly transformed clinical practice, with an increasing proportion of patients presenting advanced age, frailty, multimorbidity, and functional vulnerability. Conventional cardiology models, largely derived from younger and selected populations, often fail to adequately address the complexity of cardiovascular care in older adults. Despite the growing development of cardiogeriatrics, the core competencies required for contemporary geriatric cardiology practice remain insufficiently defined. **Methods**: This narrative review synthesises evidence from cardiology, geriatrics, heart failure, and the palliative care literature, complemented by clinical expertise in integrated cardiogeriatric care pathways, to identify key competencies relevant to the care of older adults with cardiovascular disease. **Results**: Four major domains of geriatric cardiology competencies were identified: (1) advanced cardiovascular expertise adapted to ageing physiology, frailty, and multimorbidity; (2) integration of comprehensive geriatric assessment into cardiovascular decision-making; (3) a dedicated cardiogeriatric communication mindset supporting shared decision-making under prognostic uncertainty; and (4) system-based competencies focused on multidisciplinary coordination, care transitions, and therapeutic proportionality. **Conclusions**: Defining the core competencies of the geriatric cardiologist is essential to addressing the clinical and organisational challenges of an ageing cardiovascular population. This framework provides a pragmatic foundation for clinical practice, education, and future research, supporting integrated cardiogeriatric care models aligned with patient-centred outcomes.

## 1. Introduction

The demographic ageing of the cardiovascular population represents one of the most profound transformations in contemporary cardiology. Advances in prevention and treatment have substantially improved survival, resulting in a growing number of patients living to advanced age with chronic cardiovascular disease. Older adults now constitute the majority of patients affected by heart failure (HF), atrial fibrillation, and valvular heart disease, conditions that account for a large proportion of cardiovascular morbidity and healthcare utilisation worldwide [1].

Cardiovascular disease in later life rarely occurs in isolation. Older patients frequently present with frailty, multimorbidity, cognitive impairment, functional dependence, and social vulnerability, all of which substantially modify disease trajectories, treatment tolerance, and outcomes [2,3]. Frailty, in particular, has emerged as a key determinant of prognosis in HF and other cardiovascular conditions, independently associated with increased mortality, hospitalisation, and poorer quality of life [2,3]. In HF, frailty exerts a strong and independent impact on prognosis, hospitalisation risk, and quality of life, beyond conventional cardiac severity markers. Recent studies emphasise that a multidimensional assessment integrating functional, cognitive, nutritional, and social domains is essential to adequately characterise vulnerability and guide therapeutic decision-making in older HF patients [4,5]. As such, frailty assessment has become a central element of contemporary cardiovascular decision-making in ageing populations.

Despite this evolving clinical reality, traditional cardiology models remain largely shaped by evidence derived from randomised clinical trials conducted in younger and highly selected populations. Older adults—particularly those with frailty, multiple comorbidities, or cognitive impairment—are consistently underrepresented in cardiovascular clinical trials, limiting the external validity of guideline recommendations for real-world elderly populations [6]. Consequently, guideline-directed medical therapies (GDMT), while effective at a population level, may be difficult to apply uncritically in very old patients with limited physiological reserve and competing risks [7]. In line with this evolving clinical complexity, recent European Society of Cardiology (ESC) guidelines on HF, valvular heart disease, atrial fibrillation, and cardiovascular prevention increasingly emphasise the importance of frailty assessment, geriatric syndromes, and individualised decision-making in older patients [1].

Clinicians are therefore increasingly confronted with complex situations in which therapeutic decisions must balance expected cardiovascular benefit against treatment burden, adverse effects, and patient priorities. In advanced cardiovascular disease, especially HF, this complexity often extends beyond disease modification alone and includes functional preservation, symptom control, and alignment of care with individual goals and values [8].

In response to these challenges, cardiogeriatrics has emerged as an interdisciplinary field aiming to bridge cardiology and geriatrics by integrating advanced cardiovascular expertise with comprehensive geriatric assessment (CGA), shared decision-making, and coordination across care settings [9,10]. Rather than focusing exclusively on disease-centred outcomes such as survival, cardiogeriatrics emphasises functional status, quality of life, and patient-centred outcomes as central targets of care [10]. In this context, the geriatric cardiologist plays a pivotal role in cardiac rehabilitation, by integrating cardiovascular recovery with frailty assessment, comorbidity management, functional reconditioning, and realistic goal setting in very old and vulnerable patients. However, despite its growing recognition, cardiogeriatrics remains insufficiently structured as a clinical discipline. The specific competencies required to practise cardiogeriatric medicine are rarely defined explicitly, and most cardiologists acquire these skills informally, without dedicated training frameworks or shared standards [11].

In this context, the present narrative review and position paper proposes a competency-based framework outlining the core clinical, communicational, organisational, and ethical skills required for the modern geriatric cardiologist, with the aim of supporting comprehensive, patient-centred cardiovascular care in ageing populations [12].

## 2. Materials and Methods

### 2.1. Study Design

This work was conceived as a narrative review and position paper reflecting the perspective of a limited group of physicians with shared clinical expertise in cardiogeriatric care. It does not represent a formal consensus statement or guideline, but rather a conceptual framework intended to stimulate discussion, education, and future research in geriatric cardiology. Given the conceptual and integrative nature of the research question, a narrative approach was selected to allow synthesis across heterogeneous domains, including cardiology, geriatrics, HF management, communication science, and healthcare organisation.

### 2.2. Literature Search and Sources

A non-systematic literature search was conducted using PubMed/MEDLINE, focusing on English-language publications relevant to cardiovascular care in older adults. Search terms included combinations of geriatric cardiology, HF, older adults, frailty, multimorbidity, shared decision-making, care transitions, polypharmacy, and palliative care. Additional sources were identified through manual screening of reference lists from international cardiology and geriatrics guidelines, consensus documents, and seminal narrative reviews.

Given the conceptual and competency-based objectives of this work, a systematic review methodology was not pursued. Instead, a narrative approach was selected to allow integration of heterogeneous evidence across clinical, organisational, and ethical domains. While this approach may introduce selection bias, it was considered appropriate to support the development of a pragmatic and clinically oriented competency framework rather than to quantify intervention effects.

Priority was given to international guidelines and position statements, high-quality reviews and expert consensus documents, observational and interventional studies involving older or frail cardiovascular populations, and publications addressing multidisciplinary care models and communication strategies in complex clinical settings.

To enhance transparency regarding the evidence underpinning the proposed competencies, Table 1 summarises representative and non-exhaustive studies supporting each cardiogeriatric competency domain.

### 2.3. Conceptual Framework Development

The competency framework proposed in this article was developed through an iterative synthesis of the selected literature combined with expert clinical experience in cardiogeriatric care pathways. Rather than aggregating quantitative outcomes, the analysis focused on identifying recurring themes, skill domains, and decision-making challenges consistently reported across studies and guidelines.

Identified competencies were organised into thematic domains reflecting real-world clinical practice, including cardiovascular expertise adapted to ageing, integration of geriatric assessment, communication skills, system-based coordination, and ethical decision-making.

### 2.4. Scope and Limitations

This review did not aim to provide an exhaustive or systematic appraisal of all available evidence, nor to quantify effect sizes associated with specific interventions. Instead, it sought to offer a pragmatic and clinically applicable framework to support cardiologists involved in the care of older adults. As a narrative review and position paper, this manuscript reflects the interpretation and clinical experience of a limited group of clinicians and does not aim to provide consensus-level or prescriptive recommendations.

## 3. From Disease-Centred Cardiology to Patient-Centred Cardiogeriatrics

Despite its growing recognition, cardiogeriatrics remains insufficiently structured as a clinical discipline. Recent reviews have highlighted that cardiogeriatrics is still heterogeneously implemented across healthcare systems, with wide variability in how geriatric principles are integrated into cardiovascular practice [13]. This lack of formalisation partly reflects the absence of clearly defined competencies and standardised training pathways for cardiologists involved in the care of older adults.

The clinical challenge is further amplified by the rapid growth of the “oldest old” population. Contemporary epidemiological analyses emphasise that patients aged ≥85 years represent a fast-expanding subgroup with cardiovascular disease, characterised by high prevalence of frailty, multimorbidity, and functional vulnerability, and by outcomes that differ substantially from those observed in younger cohorts [14]. Frailty has emerged as a central determinant of prognosis in this population, with recent mechanistic and clinical studies demonstrating its strong association with adverse cardiovascular outcomes, hospitalisation, and loss of functional independence [15].

In parallel, there is increasing recognition that systematic frailty assessment and CGA can meaningfully inform cardiovascular decision-making. Recent work has shown that structured geriatric assessment improves risk stratification and care planning in elderly patients undergoing cardiovascular interventions, including transcatheter valve procedures [16]. Similar findings have been reported in chronic HF populations, where frailty and multimorbidity strongly influence therapeutic tolerance and outcomes, independent of conventional cardiac parameters [17,18].

Despite this growing body of evidence, the integration of cardiogeriatric principles into routine cardiovascular care remains inconsistent. Contemporary clinical reviews continue to underline gaps between available evidence and real-world practice, particularly regarding treatment individualisation, deprescribing, and coordination of care in older HF patients [19]. Moreover, recent position papers have highlighted the need to reassess cardiovascular risk management strategies in older adults, moving beyond disease-centred approaches toward models that incorporate functional status, patient priorities, and life expectancy [20].

Implementation studies further suggest that barriers to cardiogeriatric practice include limited clinician training, time constraints, and insufficient integration of frailty assessment into cardiology workflows [21]. Emerging data indicate that multiprofessional, integrated interventions may help address these challenges, supporting more coherent and patient-centred care for older adults with cardiovascular disease [22].

Together, these observations underscore the need to move beyond fragmented recommendations toward a unified, competency-based framework capable of guiding cardiologists in the comprehensive management of older cardiovascular patients.

**Figure 1:**
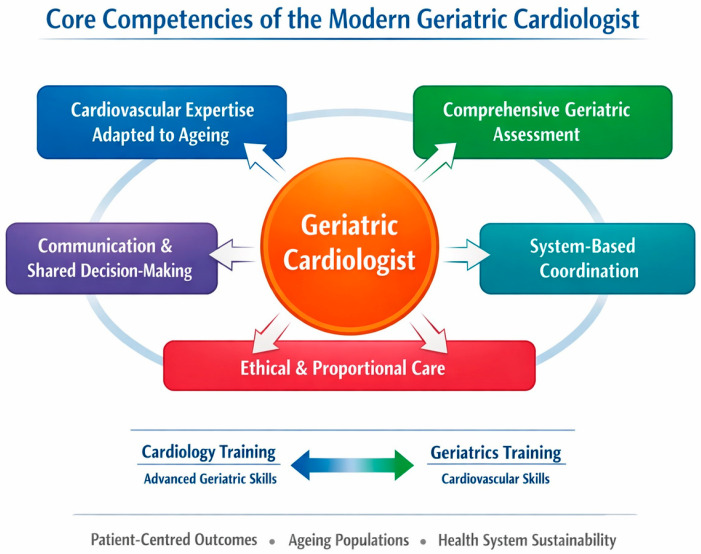
Core competencies of the modern geriatric cardiologist. Legend: Core competency domains of contemporary geriatric cardiology, including cardiovascular expertise adapted to ageing, comprehensive geriatric assessment, communication and shared decision-making skills, system-based coordination, and ethical reasoning.

To synthesise these conceptual foundations, Figure 1 illustrates the core competency domains required for modern geriatric cardiology practice, integrating cardiovascular expertise, geriatric assessment, communication, system-based coordination, and ethical reasoning.

## 4. Core Clinical Competencies in Geriatric Cardiology

### 4.1. Cardiovascular Expertise Adapted to Ageing

The geriatric cardiologist must maintain a high level of cardiovascular expertise while adapting diagnostic and therapeutic strategies to the physiological changes associated with ageing. Age-related alterations in cardiovascular reserve, renal function, autonomic regulation, and body composition significantly influence pharmacokinetics and pharmacodynamics, increasing interindividual variability in treatment response and susceptibility to adverse drug reactions [23,24].

In HF, clinical competence extends beyond the prescription of GDMT to include careful assessment of tolerability, prioritisation of therapeutic objectives, and dynamic adjustment of treatment intensity. In older patients, treatment decisions frequently involve balancing the expected benefits of disease-modifying therapies against risks such as hypotension, renal deterioration, electrolyte disturbances, and worsening functional status [23,25,26]. Frailty and multimorbidity further compound this complexity and are strongly associated with higher rates of hospitalisation, adverse events, and mortality, independent of traditional cardiac severity markers [24,27]. In this context, recent evidence highlights that frailty is a multidimensional construct requiring systematic assessment across physical, cognitive, nutritional, and social domains. Such multidomain evaluation provides prognostic information complementary to left ventricular ejection fraction, biomarkers, or symptom severity, and is increasingly recognised as a cornerstone of individualised HF management in older adults [4,5].

Similar considerations apply to atrial fibrillation management, where anticoagulation strategies must integrate thromboembolic and bleeding risks alongside cognitive status, fall risk, and adherence capacity, as well as to valvular heart disease, where procedural decisions require appraisal of frailty, comorbidities, life expectancy, and anticipated functional benefit [25]. Recent ESC guidelines on valvular heart disease have lowered the age threshold for considering transcatheter aortic valve implantation from 75 to 70 years, further emphasising that chronological age alone should not drive procedural decisions. In this evolving context, frailty and multidimensional assessment play a central role in patient selection and expected functional benefit [28]. Cardiovascular expertise in geriatric cardiology therefore includes not only technical and pharmacological knowledge, but also longitudinal monitoring, early identification of intolerance, and timely treatment de-escalation when the burden of therapy outweighs expected benefit [26,27].

Beyond HF, cardiogeriatric expertise is essential across a wide range of cardiovascular conditions. In coronary artery disease, the geriatric cardiologist contributes to balancing invasive versus conservative strategies by integrating frailty, cognitive status, bleeding risk, and life expectancy into decision-making. In valvular heart disease, particularly aortic stenosis (AS), cardiogeriatric assessment supports patient selection for transcatheter or surgical interventions by aligning procedural benefit with functional prognosis and patient goals. In peripheral arterial and aortic diseases, management strategies must account for functional limitation, polyvascular disease, and competing risks, often favouring personalised and proportionate approaches. Finally, within HF itself, the geriatric cardiologist plays a key role in differentiating phenotypes, particularly HFrEF versus HFpEF, and in adapting guideline-directed therapies to tolerance, comorbidities, and patient priorities.

To illustrate how cardiogeriatric competencies translate into clinical decision-making, Table 2 contrasts standard guideline-based approaches with cardiogeriatric strategies in selected cardiovascular scenarios frequently encountered in very old patients.

Cardiovascular risk factors represent another key domain in which cardiogeriatric expertise is essential. In older adults, the management of hypertension, diabetes mellitus, and dyslipidaemias requires individualised targets that balance cardiovascular prevention with functional status, treatment burden, and risk of adverse events. The geriatric cardiologist plays a central role in adapting preventive strategies to frailty, multimorbidity, and limited physiological reserve.

In particular, diabetes mellitus poses specific challenges in older patients, given its close interaction with cardiovascular disease, functional decline, and cognitive impairment. The geriatric cardiologist contributes to the early identification and management of cardiovascular complications of diabetes, including diabetic cardiomyopathy, and to the integration of cardiac imaging, metabolic control, and therapeutic strategies within a comprehensive, patient-centred approach [29].

### 4.2. Integration of Comprehensive Geriatric Assessment

A defining competency of geriatric cardiology is the systematic integration of CGA into cardiovascular decision-making. Key domains include frailty, functional status, cognition, nutrition, mobility, mood, and social support, all of which substantially influence prognosis, treatment tolerance, and care trajectories in older cardiovascular patients [24,25].

Frailty can be assessed using different validated tools depending on clinical context, including phenotype-based instruments (e.g., Fried frailty phenotype), deficit-accumulation approaches (e.g., Frailty Index), and short screening tools frequently used in cardiology such as the Clinical Frailty Scale or gait speed. In cardiovascular care, pragmatic tools that are rapid, reproducible, and predictive of outcomes are often favoured.

Beyond frailty, multidimensional assessment is particularly useful in situations of therapeutic uncertainty, discordance between symptom burden and cardiac severity, recurrent hospitalisations, cognitive impairment, or suspected functional decline. In these contexts, comprehensive geriatric assessment helps clarify prognosis, identify reversible contributors, and guide individualised decision-making.

Frailty assessment provides critical prognostic information that complements conventional cardiovascular risk stratification. Frail patients are at increased risk of procedural complications, hospitalisation, and functional decline, even when traditional cardiac parameters appear stable [24,27]. Impairments in activities of daily living, recurrent falls, malnutrition, or cognitive dysfunction often precede adverse outcomes and may signal vulnerability that is not captured by disease-specific severity scores [25].

Recent studies have demonstrated that structured geriatric assessment improves risk stratification and supports more individualised care planning in elderly patients undergoing cardiovascular interventions [16,23]. In chronic HF populations, geriatric assessment has been shown to inform therapeutic prioritisation, anticipate intolerance to aggressive treatment, and guide integration of supportive and multidisciplinary care [23,27].

Incorporating these elements allows the geriatric cardiologist to better anticipate complications, tailor follow-up strategies, and align cardiovascular treatments with realistic and meaningful goals of care, ensuring that clinical decisions reflect not only disease characteristics but also functional priorities, patient preferences, and anticipated quality of life [24,26].

## 5. Communication Competencies and Shared Decision-Making

### 5.1. Shared Decision-Making in Complex Situations

Cardiovascular care in older adults is frequently characterised by prognostic uncertainty, competing risks, and trade-offs between longevity, symptom control, functional independence, and treatment burden. In this context, shared decision-making has been increasingly recognised as a core component of high-quality cardiovascular care rather than a supplementary communication skill [30]. Recent scientific statements emphasise that shared decision-making is particularly relevant in complex clinical situations where evidence is limited or heterogeneous, as is often the case in very old or frail patients [30,31].

Shared decision-making in cardiogeriatrics requires the ability to communicate potential benefits and harms of interventions in a balanced, comprehensible, and individualised manner. This includes adapting information to patients’ cognitive and sensory capacities, exploring personal values and preferences, and explicitly addressing uncertainty [31,32]. Rather than focusing exclusively on survival or disease-specific endpoints, discussions frequently prioritise symptom burden, quality of life, maintenance of autonomy, and acceptable levels of treatment-related burden, outcomes that older patients consistently identify as meaningful [30,31].

Importantly, shared decision-making in older cardiovascular patients is not a single event but an iterative process embedded in longitudinal care. As disease trajectories evolve and functional status changes, preferences may shift, requiring regular reassessment of goals of care and treatment priorities. Recent reviews underline that failure to revisit decisions over time may contribute to overtreatment, decisional conflict, and misalignment between care delivered and patient goals [33,34].

### 5.2. The Cardiogeriatric Communication Mindset

Beyond individual decision-making encounters, cardiogeriatric practice relies on a specific communication mindset shaped by chronic disease trajectories, uncertainty, and the progressive nature of ageing. This mindset is characterised by progressive temporality, repetition, and continuous adaptation to cognitive, sensory, and emotional limitations commonly encountered in older adults [33,35].

Key elements include translating complex cardiovascular information into meaningful, patient-centred concepts, avoiding technical jargon, and framing medical decisions in relation to daily functioning and lived experience. Revisiting goals of care over time is essential, particularly following sentinel events such as hospitalisation, functional decline, or treatment intolerance. Recent qualitative and implementation studies suggest that explicitly acknowledging uncertainty and normalising its presence may reduce anxiety and foster realistic expectations among patients and families [35,36,37].

This communication approach supports sustained alignment between clinical decisions and evolving patient priorities and facilitates anticipation of future care needs, including advance care planning. Emerging data indicate that structured communication strategies integrated into cardiovascular care pathways may improve patient understanding, reduce decisional regret, and support more coherent transitions between curative, supportive, and palliative approaches when appropriate [36,37,38,39]. In advanced or chronic cardiovascular disease, the cardiogeriatric communication mindset therefore plays a central role in ensuring that care remains proportionate, coherent, and consistent with patient values across the disease trajectory.

## 6. System-Based and Organisational Competencies

### 6.1. Multidisciplinary Coordination

Older cardiovascular patients frequently require coordinated input from multiple professionals, including geriatricians, specialist and advanced practice nurses, physiotherapists, pharmacists, primary care physicians, and, in advanced stages, palliative care teams. Contemporary HF guidelines increasingly recognise that optimal outcomes in older adults depend not only on pharmacological optimisation but also on structured, multidisciplinary care models capable of addressing multimorbidity, functional impairment, and social vulnerability [40].

In this context, the geriatric cardiologist plays a central role in orchestrating multidisciplinary coordination. This role extends beyond referral or consultation and involves actively integrating inputs from different disciplines into a coherent and prioritised care plan. Recent clinical trials and implementation studies have shown that structured coordination, early optimisation of care after hospitalisation, and clear clinical accountability are associated with improved outcomes and reduced rehospitalisation in HF populations, including older and more complex patients [41,42].

Effective multidisciplinary coordination also includes integration of non-pharmacological interventions such as physical rehabilitation, which has been shown to improve functional capacity and quality of life in older patients hospitalised for HF [43]. Moreover, emerging care models specifically targeting older, multimorbid HF patients highlight the importance of coordinated, patient-centred pathways that bridge cardiology, geriatrics, and community-based services [44].

### 6.2. Care Transitions and Continuity

Transitions between care settings—such as discharge from hospital to home, rehabilitation units, or long-term care facilities—represent periods of heightened vulnerability for older cardiovascular patients. These transitions are associated with increased risks of medication discrepancies, functional decline, and early readmission, underscoring the need for structured transition-of-care strategies [40].

Key organisational competencies include comprehensive discharge planning, early post-discharge follow-up, and proactive identification of patients at high risk of deterioration. Evidence from recent trials and meta-analyses suggests that structured follow-up programmes and coordinated transitional care can reduce rehospitalisation and improve continuity, particularly when initiated early after discharge [41,42].

Digital tools such as telemonitoring and remote patient monitoring may further support continuity of care by enabling early detection of clinical deterioration and facilitating communication between hospital-based teams and community providers. Recent meta-analyses indicate that telemonitoring can reduce HF-related hospitalisations, particularly when programmes are embedded within integrated care pathways rather than implemented as standalone technological solutions [45,46,47].

Finally, effective care transitions also require attention to medication reconciliation and coordination with post-acute and community services. Recent studies have highlighted the persistence of medication discrepancies following discharge from post-acute care facilities, reinforcing the need for systematic coordination across settings and clear communication between providers [48,49].

Together, these organisational competencies support the development of integrated cardiogeriatric care pathways that prioritise continuity, proportionality of interventions, and alignment of healthcare delivery with the complex and evolving needs of older cardiovascular patients.

## 7. Ethical and Professional Competencies

Ethical decision-making is intrinsic to cardiogeriatric practice, given the high prevalence of multimorbidity, frailty, and limited physiological reserve among older cardiovascular patients. Clinical situations involving treatment limitation, deprescribing, or de-escalation of care are common, particularly in advanced disease stages where the balance between benefit and harm becomes increasingly uncertain [50].

Frailty assessment may also inform deprescribing decisions, including the discontinuation or non-initiation of costly disease-modifying therapies when the expected clinical benefit is limited by reduced life expectancy, high treatment burden, or competing risks. In this context, frailty supports therapeutic proportionality by aligning resource-intensive treatments with realistic patient-centred outcomes.

The concept of therapeutic proportionality provides a central ethical framework for cardiogeriatric decision-making. Rather than focusing solely on disease severity or guideline eligibility, therapeutic proportionality emphasises the balance between expected clinical benefit, treatment burden, potential adverse effects, and the patient’s individual goals and values. Evidence from clinical and ethical studies supports that reconsideration or withdrawal of long-term cardiovascular therapies may be appropriate when treatments no longer contribute to meaningful patient-centred outcomes [50,51].

Professional competence in cardiogeriatrics therefore includes the ability to recognise when cardiovascular interventions may become non-beneficial or even harmful, and to initiate timely discussions about treatment adaptation or de-escalation. This includes anticipatory conversations about future care preferences and the integration of serious illness care principles into cardiovascular management. Such approaches have been increasingly advocated within cardiology as essential to ensure that care remains aligned with patient values, while supporting patients and families in navigating complex decisions across the disease trajectory [52].

## 8. Training, Education, and Implementation

Despite the growing clinical need for cardiogeriatric expertise, formal training pathways in cardiogeriatrics remain limited and heterogeneous across healthcare systems. Multiple authors have highlighted that contemporary cardiology training is still largely oriented toward disease-centred models derived from younger populations, leaving clinicians insufficiently prepared to manage the complexity of older adults with multimorbidity, frailty, and functional vulnerability [27].

Developing structured educational programmes is therefore essential to support the dissemination and sustainability of cardiogeriatric practice. This includes integrating geriatric cardiology into undergraduate and postgraduate cardiology curricula, promoting interdisciplinary training modules involving geriatrics, nursing, rehabilitation, and palliative care, and adopting competency-based frameworks that reflect real-world clinical challenges. The systematic incorporation of frailty assessment and geriatric principles into cardiovascular training has been identified as a key step to improve clinical decision-making and care individualisation in older patients [53].

Beyond individual education, successful implementation of cardiogeriatrics also requires institutional recognition and organisational support. Recent reviews emphasise that recognising cardiogeriatrics as a distinct and legitimate field within cardiovascular medicine may facilitate the development of dedicated care pathways, allocation of appropriate resources, and integration into quality improvement and accreditation processes [13]. At a system level, embedding cardiogeriatric competencies into professional standards and healthcare structures may help ensure that ageing cardiovascular populations receive care that is both evidence-informed and aligned with their complex clinical, functional, and social needs.

## 9. Future Perspectives

Future research should move beyond descriptive analyses to evaluate the impact of cardiogeriatric competencies on outcomes that matter most to older adults, including symptom burden, functional status, quality of life, and goal-concordant care, alongside traditional endpoints such as hospitalisation and mortality. In this context, predefined key performance indicators may include rates of functional decline, unplanned hospitalisation, medication-related adverse events, documentation of shared decision-making, and alignment between delivered care and patient goals. Particular attention should be paid to very old patients (≥85 years), who remain underrepresented in cardiovascular trials despite representing a rapidly growing population with advanced disease and complex care needs.

At the system level, studies should assess the effectiveness of integrated cardiogeriatric care models, including their impact on healthcare utilisation, continuity of care, and avoidance of non-beneficial interventions. Implementation research will be essential to translate cardiogeriatric competencies from conceptual frameworks into routine clinical practice and to identify context-specific facilitators and barriers across healthcare systems.

Beyond these organisational considerations, the development of cardiogeriatric expertise raises broader questions regarding workforce training and professional boundaries. In addition to strengthening geriatric competencies within cardiology, future perspectives should consider the reciprocal development of advanced cardiovascular expertise within geriatric medicine. In many healthcare systems, geriatricians provide longitudinal care to large populations of very old and frail patients with limited access to specialised cardiology services, potentially exposing them to underdiagnosis or suboptimal management of cardiovascular disease.

Given that HF, valvular heart disease, and atrial fibrillation predominantly affect older adults, empowering geriatricians with dedicated cardiology skills—including echocardiography, haemodynamic interpretation, and optimisation of HF therapies—may represent a pragmatic and sustainable model of care. Such training pathways could foster the emergence of dual-profile cardiogeriatricians, able to identify cardiovascular disease earlier, participate fully in therapeutic decision-making, and move beyond a purely consultative role.

This model may improve continuity of care, enhance professional engagement, and ensure that complex cardiovascular decisions in older patients are informed by both geriatric and cardiological expertise. In this perspective, cardiogeriatrics may evolve from an interface between specialties into a distinct field defined by hybrid expertise and shared responsibility for the management of age-related cardiovascular disease, while allowing cardiologists to increasingly focus on younger populations and highly specialised interventional care [3,13,18,23].

## 10. Conclusions

The ageing of cardiovascular populations challenges traditional disease-centred cardiology and calls for a redefinition of clinical competencies adapted to later life. Cardiovascular care in older adults is shaped by the complex interplay between cardiovascular disease, frailty, multimorbidity, functional vulnerability, and heterogeneous patient priorities, which cannot be fully addressed by conventional guideline-based approaches alone.

This position paper proposes a competency-based framework for modern geriatric cardiology practice, integrating cardiovascular expertise adapted to ageing, CGA, a dedicated communication mindset supporting shared decision-making under uncertainty, system-based coordination across care settings, and ethical reasoning grounded in therapeutic proportionality. Together, these domains provide a foundation for patient-centred, proportionate, and coordinated cardiovascular care in ageing populations.

Beyond defining competencies, this framework also invites reflection on how cardiogeriatric expertise should be developed within healthcare systems. In addition to equipping cardiologists with geriatric skills, strengthening cardiovascular competencies within geriatric medicine itself may represent a complementary and sustainable strategy, allowing geriatricians to assume an active role in the diagnosis, management, and decision-making of age-related cardiovascular disease.

Several limitations should be acknowledged. This work is a narrative review and position paper and does not provide a systematic or quantitative evaluation of the evidence. Moreover, the proposed framework remains conceptual and has not yet been prospectively validated, and its implementation may vary across healthcare systems.

Future research should evaluate the impact of cardiogeriatric competencies and integrated care models on patient-centred outcomes, healthcare utilisation, and care quality, with particular focus on very old patients who remain underrepresented in clinical trials. Implementation and health services research will be essential to support the translation of cardiogeriatric competencies into routine cardiovascular practice and healthcare policy.

## Figures and Tables

**Table 1:** Key evidence supporting cardiogeriatric competency domains.

Competency Domain	Key Evidence Type	Representative References
Frailty & HF	Reviews/consensus	Eur. J. Heart Fail., ESC HF
CGA integration	Observational/expert	JACC, J. Clin. Med.
Communication	Scientific statements	Circulation, JAMA
Care coordination	Trials/meta-analyses	Lancet, Eur. J. HF

**Table 2:** Comparison between standard cardiology and cardiogeriatric approaches in selected cardiovascular conditions among very old patients.

Clinical Scenario	Standard Guideline Approach	Cardiogeriatric Approach
HF ≥85 years	GDMT escalation	Prioritisation, tolerance, function
Severe AS	Procedural eligibility	Frailty, life expectancy, goals
Atrial Fibrillation	Stroke prevention focus	Falls, cognition, adherence

## Data Availability

The datasets used and/or analysed during the current study are available from the corresponding author (R.E.) on reasonable request. Due to patient privacy restrictions, data are not publicly available.

## Abbreviations

The following abbreviations are used in this manuscript:

AF	Atrial Fibrillation
AS	Aortic Stenosis
CGA	Comprehensive Geriatric Assessment
ESC	European Society of Cardiology
GDMT	Guideline-Directed Medical Therapy
HF	Heart Failure
HFpEF	Heart Failure with Preserved Ejection Fraction
HFrEF	Heart Failure with Reduced Ejection Fraction
